# Standardization of international diverticular disease definitions

**DOI:** 10.1111/codi.70585

**Published:** 2026-08-12

**Authors:** A. L. Fahey, Z. M. Khan, S. Lawday, M. Lee, D. Vimalachandran, A. T. Hawkins

**Affiliations:** ^1^ Vanderbilt University School of Medicine Nashville Tennessee USA; ^2^ Department of General Surgery Liverpool University Foundation Hospital Trusts Liverpool UK; ^3^ Centre for Surgical Research University of Bristol Bristol UK; ^4^ Department of Applied Health Sciences, School of Health Sciences, College of Medicine and Health University of Birmingham Birmingham UK; ^5^ Institute of Systems, Molecular and Integrative Biology, University of Liverpool Liverpool UK; ^6^ Section of Colon and Rectal Surgery Vanderbilt University Medical Center Nashville Tennessee USA

**Keywords:** consensus definitions, diverticular disease, survey

## Abstract

**Aim:**

The purpose of this study was to achieve international expert consensus on definitions describing diverticular disease and its associated disease processes to establish a standardized nomenclature applicable to both clinical practice and research within colorectal surgery.

**Methods:**

This was a cross‐sectional survey. The research team developed initial definitions through a search of online diverticular disease literature. Definitions were refined through extensive beta testing, resulting in definitions for the survey. The survey was distributed through snowball methodology to global experts who have experience treating diverticular disease. Participants were given the option to provide feedback via free text on a definition if they disagreed with it. Based on prior definition consensus studies, an 80% agreement threshold was used to reach consensus. Demographic data was also collected from respondents.

**Results:**

Two hundred and four completed responses were received following survey distribution. Based on the survey results, each proposed definition met consensus agreement. The median years of practice among respondents were 12 years. Most identified as surgical physicians (*n* = 178, 87.3%), while 23 (11.3%) identified as medical physicians. The most represented countries among respondents were the United States (*n* = 100, 49.0%), the United Kingdom (*n* = 65, 31.9%) and Canada (*n* = 13, 6.4%).

**Conclusion:**

The study revealed consensus from various healthcare providers across the world. Some definitions had higher agreement than others, revealing a need to investigate the nuances of certain definitions further. These standardizations of common diverticulitis terms will enhance research by increasing generalizability, as well as improve clinical communication between patients and providers with consistent definitions.

## INTRODUCTION

Left‐sided diverticular disease is a common colorectal condition that ranges from uncomplicated diverticulosis through to perforation of diverticula and faecal peritonitis. These presentations include acute diverticulitis, chronic diverticulitis, uncomplicated diverticulitis, complicated diverticulitis, as well as less common variants such as segmental colitis associated with diverticulitis (SCAD) and symptomatic uncomplicated diverticular disease (SUDD) [1, 2]. Although diverticulosis is asymptomatic, diverticulitis and its sequelae account for a substantial proportion of healthcare utilization, annually accounting for more than 2.7 million outpatient visits, 200,000 inpatient admissions and an economic burden of $2 billion in the United States alone [1]. Additionally, the increasing prevalence of diverticular disease globally makes it one of the most commonly encountered colorectal conditions worldwide, with recent literature estimating 60% of individuals over the age of 60 having diverticula and 4% of those individuals experiencing diverticulitis [1, 3].

Despite its clinical importance, terminology describing diverticular disease remains inconsistent. Different disease presentations have evolved, yet there has been no consistent agreement on definitions. Consequently, similar clinical presentations may be categorized differently depending on whether symptoms, radiological findings, operative assessments or histopathology are used. While studies of diverticular disease propose definitions for the investigated condition [2, 4, 5, 6, 7], these are often study‐specific, with no uniform consensus being reached on defining diverticular disease and its complications.

The absence of standardized definitions has important implications for both research and clinical care. Variation in terminology contributes to heterogeneity in reported research and limits the ability to synthesize evidence across studies [8, 9]. Multiple studies exist in the current surgical literature that have utilized survey‐based approaches to achieve consensus definitions, including a study that defined anastomotic leakage in colorectal surgery through a modified Delphi survey study [10], as well as another that utilized surveys to define ‘major surgeries’ [11]. For colorectal surgeons, disease classification may influence treatment selection, timing of operative intervention and patient counselling, underscoring the need for clear, reproducible disease definitions [12].

Standardized definitions are therefore essential to improve the reporting, interpretation and generalizability of diverticular disease research. This study aimed to achieve international expert consensus on definitions describing diverticular disease and its associated disease processes to establish a standardized nomenclature applicable to both clinical practice and research within colorectal surgery.

## MATERIALS AND METHODS

This study utilized a consensus‐based approach to inform disease descriptors. This approach has been used in other disease processes to establish outcomes and descriptors [13, 14]. Applying a similar framework will ensure terminology reflects contemporary clinical practice. To obtain initial definitions on the various entities within diverticular disease, PubMed searches were conducted by three members of the research team (AF, SL, ZK) to review available definitions within the literature. Based on initial literature review, definitions were extracted for nine terms, including, ‘colonic diverticulum’, ‘colonic diverticulosis’, ‘acute left‐sided diverticulitis’, ‘complicated diverticulitis’, ‘uncomplicated diverticulitis’, ‘chronic recurrent diverticulitis’, ‘chronic persistent diverticulitis’, ‘symptomatic Uncomplicated Diverticular Disease’ and ‘segmental colitis associated with diverticulosis’. Proposed definitions were reviewed by three junior members of the research team, then presented to the full research group for feedback. Definitions were refined and finalized for use in the survey.

Consensus‐driven standardization has improved research quality and reporting in other complex disease processes, allowing clearer comparison of outcomes. Previous methodological studies have demonstrated that structured consensus approaches incorporating systematic literature review and multidisciplinary expert input can successfully establish uniform definitions [13, 14]. Applying a similar framework to diverticular disease may provide a shared terminology that better reflects contemporary clinical practice and supports future research.

The Checklist for Reporting Results of Internet E Surveys (CHERRIES) was adhered to during the survey design process in order to ensure the quality of the web‐based survey [15]. The survey was designed to be distributed to health professionals who treat left‐sided diverticular disease. The survey was pre‐tested on researchers who designed it for usability and technical functionality.

Recruitment occurred via snowball methodology, a recruitment technique wherein social networks of colleagues are utilized to increase outreach [16]. Additionally, the survey was sent to associated surgical departments and circulated to professional organizations, including the Association of Surgeons of Great Britain, the Association of Coloproctology of Great Britain, the European Society of Coloproctology and the American Society of Colon and Rectal Surgeons.

This study utilized the web‐based survey website Research Electronic Data Capture (REDCap) to collect responses from various healthcare professionals. This website allows for simple survey design with integrated data collection and export instruments for survey responses.

Upon clicking on the open survey link, information was provided on the length of the survey, the purpose of the study, and how the results would be used. The survey was voluntary to complete with no incentives offered. Personal data such as years in practice, geographical location and profession were collected and stored securely in line with General Data Protection Regulation (GDPR). This data was to be used to either ensure diverse representation between medical and surgical practitioners or to highlight the future need to garner further input from underrepresented groups following the conclusion of this study. View rate, participation rate and completion rate were tracked, and the survey only allowed completion once, which was tracked using screening questions that required answers. In total, there were 15 items, and it was mandatory to complete every item to submit and review responses.

Definitions were assessed with two binary fixed options, with responders either clicking ‘yes’ to agree or ‘no’ to disagree with each definition. If a responder clicked ‘no’, then a text box would appear below to allow for further explanation of disagreement. Based on prior consensus studies, it was determined that at least 80% agreement would need to be reached for high consensus to be achieved on each definition [17, 18]. Based on the results of the definition surveys, each proposed definition met the high consensus definition. The survey was open for a total of 23 weeks.

Demographic data was collected at the end of the survey. Information collected included years in practice, profession (medical physician, surgical physician, nursing, advanced nursing practitioner or other allied health professional), specialty (surgery, medicine, pathology or other) and country.

Only completed surveys were included in the analysis.

## RESULTS

### Demographics

In total, 204 completed responses were received following survey distribution. As the survey was voluntary to complete and widely accessible once circulated, a formal response rate could not be reported. However, a total of 268 records exist per the REDCap data collection tools. Of those, 50 were only partially filled out (81.3% completion rate). Of the remaining 218 records, 14 attempted to fill out the survey twice and were successfully screened. Overall, 204 unique completed records were collected.

The majority of respondents were in practice for >10 years (71, 6%) and predominantly surgeons (88.7%). Global representation was achieved with nearly half the respondents residing in the United States (49%).

**FIGURE 1:**
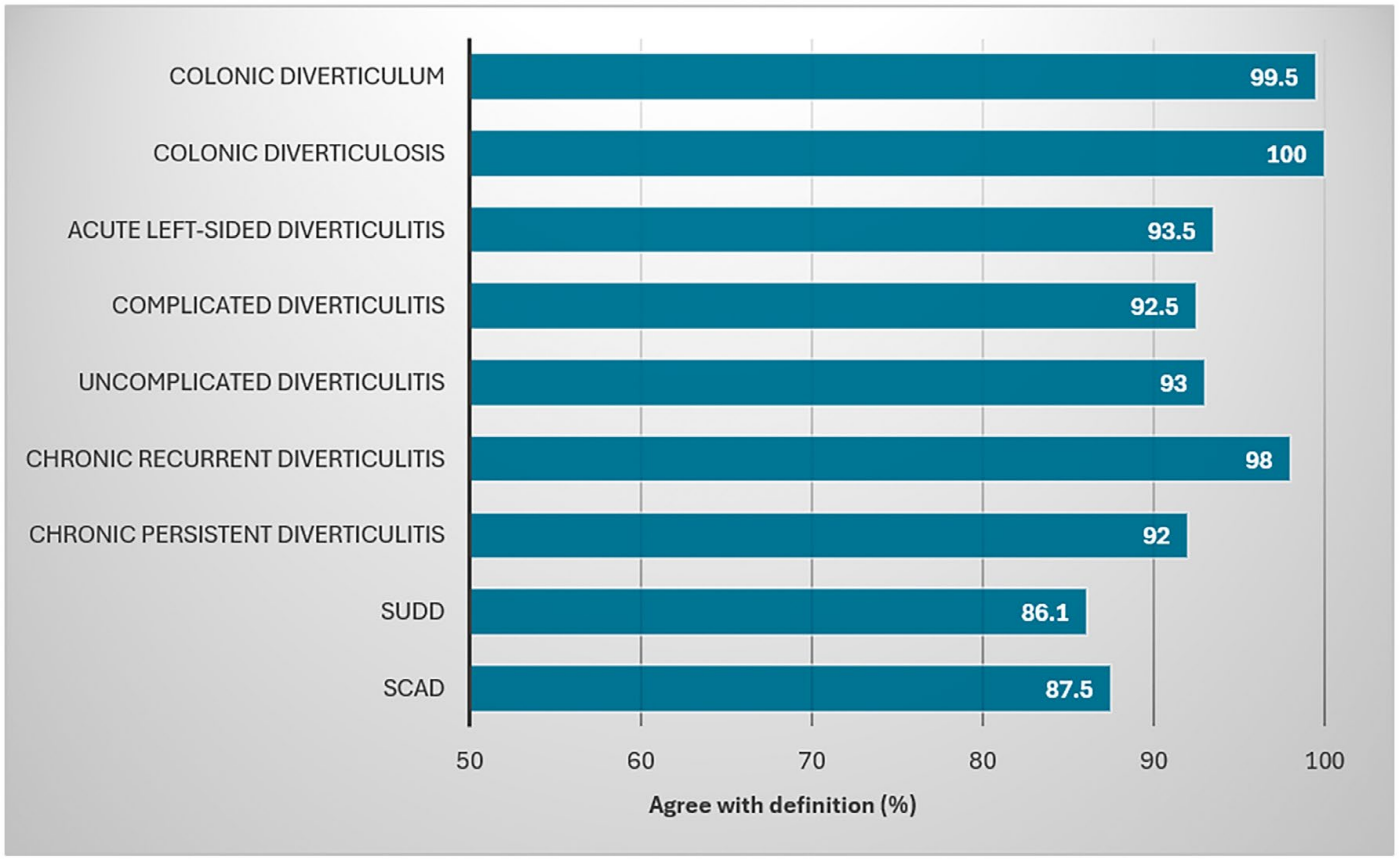
Proportion of respondents showing assent to provided definitions.

Results for each survey question on demographic data and definitions are displayed in Table 1 and Figure 1.

**TABLE 1:** Results of demographics survey (*n* = 204).

	No.	(%)
Years in Practice		
0–10	113	55.6
11–20	58	28.3
21+	33	16.1
Profession		
Surgical physician	177	86.8
Medical physician	24	11.8
Other allied health profession*	3	1.5
Specialty		
Surgery	181	88.7
Medicine	17	8.3
Other**	6	2.9
Country		
United States	100	49.0
United Kingdom	64	31.4
Canada	14	6.9
Mexico	9	4.4
Ghana	3	1.5
Italy	2	1.0
Australia	2	1.0
Denmark	2	1.0
Brazil	1	0.5
Ecuador	1	0.5
Ireland	1	0.5
Israel	1	0.5
Netherlands	1	0.5
Philippines	1	0.5
Qatar	1	0.5
Taiwan	1	0.5

*Note*: *Three respondents identified as dieticians. **Two respondents identified their specialty as ‘gastroenterology’, while one identified as a ‘dietician’.

### Definitions

#### Colonic diverticulum

‘An outpouching of mucosa and/or submucosa through the muscular layer of the bowel wall’.

This definition achieved consensus, with 99.5% agreement and only one disagreement. The disagreement was that both layers must be within the diverticulum, which is included in the definition.

#### Colonic diverticulosis

‘The presence of one or more colonic diverticula’.

This definition reached consensus with 100% agreement.

#### Acute left‐sided diverticulitis

‘Inflammation or infection within one or more diverticula of the left‐sided colon with associated symptoms including abdominal pain, fever, and/or altered bowel habits’.

This definition achieved consensus with 93.5% agreement. Among the disagreements, some respondents wanted to include objective data (laboratory findings, imaging) in the definition, while others felt that the inclusion or omission of specific symptoms was more appropriate.

#### Complicated diverticulitis

‘Inflammation of diverticula with associated abscess, uncontained perforation, stricture, bowel obstruction, or fistula’.

This definition achieved consensus with 92.5% agreement. Disagreements with this definition mostly stemmed from perforations and whether contained perforations were still considered under complicated diverticulitis.

#### Uncomplicated diverticulitis

‘Acute diverticulitis of diverticula in the absence of abscess, uncontained perforation, stricture, bowel obstruction, or fistula’.

This definition achieved consensus with 93% agreement. Disagreements were related to the previous definition and whether a contained perforation is considered uncomplicated diverticulitis or complicated diverticulitis.

#### Chronic recurrent diverticulitis

‘Intermittent episodes of acute diverticulitis separated by periods of remission’.

This definition achieved consensus with 98% agreement. Disagreements with this definition involved either the inclusion of imaging findings or the need to exclude ‘chronic’ from the definition.

#### Chronic persistent diverticulitis (smouldering diverticulitis)

‘Non‐remitting symptoms of diverticulitis, including pain, fever, and altered bowel habits’.

This definition achieved consensus with 92% agreement. Disagreements with this definition involved the inclusion of imaging findings to support the diagnosis of diverticulitis, as well as the desire to add a time period (e.g. 3 months).

#### Symptomatic uncomplicated diverticular disease (SUDD)

‘Persistent or recurrent abdominal symptoms including pain, fever, or altered bowel habits attributed to colonic diverticulosis without signs of overt diverticulitis’.

This definition achieved consensus with 86.1% agreement. Disagreements with this definition revolved around whether this disease entity exists, whether fever should be included in this definition, differentiating from irritable bowel syndrome and the need for radiological findings to rule out other causes.

#### Segmental colitis associated with diverticular disease (SCAD)

‘Nonspecific peri‐diverticular inflammation associated with rectal bleeding, abdominal pain, and/or altered bowel habits’.

This definition achieved consensus with 87.5% agreement. Disagreements with this definition were similar to those of SUDD, including whether SCAD is an individual disease entity, its similarity to other diverticular disease entities (smouldering diverticulitis, acute diverticulitis), the need to rule out other causes and disagreements around ‘peri‐diverticular’.

## DISCUSSION

This study achieved an international perspective expert consensus on standardized definitions across the spectrum of diverticular disease. All proposed definitions met predefined criteria for high agreement, with most exceeding 90% consensus, demonstrating broad professional alignment and supporting the feasibility of a unified descriptive framework for diverticular disease. This provides a foundation for a consistent definition of disease in future studies.

Near‐universal agreement was observed for the definitions of colonic diverticulum and diverticulosis. These results suggest that inconsistencies in the literature are less related to disagreement in underlying pathology, but rather variability in terminology applied to clinical presentations and disease severity. Greater discussion focused on definitions of clinical syndromes, particularly among the less common and controversial entities of symptomatic uncomplicated diverticular disease (SUDD) and segmental colitis associated with diverticular disease (SCAD). Importantly, disagreement rarely reflected rejection of the entities themselves but instead focused on diagnostic thresholds, incorporation of objective findings and classification boundaries (see Table S1).

Two recurring areas of uncertainty emerged. First, respondents differed on whether imaging or laboratory findings should be incorporated into certain definitions. It is important to recognize the increasing use of imaging and lab‐based markers in diverticular disease, particularly diverticulitis, as this reflects current contemporary practices. However, while objective criteria may improve diagnostic specificity as reliance on clinical symptoms alone can be misleading, restrictive definitions may reduce applicability across clinical and research settings. These consensus definitions therefore prioritize generalizability to clinical and research spaces while allowing study‐specific diagnostic requirements. Second, the debate over temporality in chronic diverticulitis highlights persistent ambiguity in existing classification systems and underscores the need for standardized terminology to improve comparability across studies. Consensus was lower for SUDD and SCAD, reflecting ongoing controversy over their pathophysiology and status as distinct diagnoses [4, 19]. Despite this, agreement above the consensus threshold indicates recognition of the practical need for shared terminology. Standardized definitions may facilitate future research aimed at clarifying these entities rather than perpetuating inconsistent nomenclature.

Strengths of this study include a large international respondent cohort, distribution through multiple professional organizations and use of predefined consensus methodology. Limitations include voluntary participation with potential selection bias and predominant surgical representation. It is also important to recognize the high prevalence of respondents from the United States and United Kingdom. Regional bias in patient presentations, training, collaboration and resources may skew responses to align more closely with our research group's definitions than in countries not represented in our survey. Lastly, diverticular bleeding was excluded from the initial terminology as it was felt this disease process was distinctly different from the pathophysiology affecting the other diverticular disease definitions. However, given that it is considered a part of diverticular disease by the larger medical community, further standardization of this disease process may be necessary as well.

Future work should focus on incorporating these definitions into reporting standards for clinical studies. Broad adoption will be essential to maximize impact and improve consistency in diverticular disease research; funders and specialty associations should consider implementing these in future studies.

In conclusion, consensus definitions spanning the spectrum of diverticular disease were achieved, addressing longstanding variability in terminology. Standardized definitions will enhance research comparability, provide a foundation for future outcome standardization in diverticulitis and improve clinical communications between providers and patients with concise definitions.

## AUTHOR CONTRIBUTIONS

**A. L. Fahey:** Conceptualization; writing – original draft; investigation; methodology; writing – review and editing; visualization; validation; software; formal analysis; project administration; data curation; resources. **Z. M. Khan:** Conceptualization; investigation; methodology; validation; visualization; writing – review and editing; writing – original draft; software; formal analysis; project administration; data curation; resources. **M. Lee:** Conceptualization; investigation; methodology; validation; visualization; writing – review and editing; software; formal analysis; project administration; data curation; supervision; resources. **D. Vimalachandran:** Conceptualization; investigation; methodology; validation; visualization; writing – review and editing; software; formal analysis; project administration; data curation; supervision; resources. **S. Lawday:** Conceptualization; investigation; writing – original draft; methodology; validation; visualization; writing – review and editing; software; formal analysis; project administration; data curation; resources. **A. T. Hawkins:** Conceptualization; investigation; methodology; validation; visualization; writing – review and editing; software; formal analysis; project administration; data curation; supervision; resources.

## FUNDING INFORMATION

This research did not receive any specific grant from funding agencies in the public, commercial or not‐for‐profit sectors. SL recieved a NIHR Doctoral Research Fellowship (NIHR303276). THe views expressed are those of the author(s) and not necessarily those of the NIHR or the Department of Health and Social Care.

## CONFLICT OF INTEREST STATEMENT

None.

## ETHICS STATEMENT

In accordance with institutional policy, this anonymous survey‐based study did not require Institutional Review Board approval because no identifiable personal or sensitive data were collected. Participation was voluntary, and completion of the survey implied informed consent.

## Supporting information


Table S1.

## Data Availability

The data that supports the findings of this study are available in the Supporting Information of this article.
