# Supplementary material for: Standardization of international diverticular disease definitions
Source: Colorectal Dis. 2026 Aug 12;28(8):e70585. doi: 10.1111/codi.70585 (PMC13463218; doi:10.1111/codi.70585)
Supplement: Supplementary file 1 [file CODI-28-0-s001.docx]

**Supplemental Table 1**

Representative Disagreements with each Proposed Definition

| **Colonic Diverticulum**- "An outpouching of mucosa and/or submucosa through the muscular layer of the bowel wall" | Record 208: “*both layers have to be in the diverticulum*” |
| --- | --- |
| **Colonic Diverticulosis**- "The presence of one or more colonic diverticulae" | *No disagreements* |
| **Acute Left-sided Diverticulitis**- "Inflammation or infection within one or more diverticula of the left-sided colon with associated symptoms including abdominal pain, fever, and/or altered bowel habits" | Record 46: “*need more objective data like leukocytosis, CT documentation*”  Record 90: “*Don’t think the symptoms are needed - if an asymptomatic patient had a scan showing inflamed colon, this would still be diverticulitis*”  Record 124: “*Don't always have fever ... could have pain and high inflammation markers but no fever* “  Record 156: “*No timing - rapid onset of? acute...*” |
| **Complicated Diverticulitis**- "Inflammation of diverticula with associated abscess, uncontained perforation, stricture, bowel obstruction, or fistula" | Record 109: “*Contained perforation...can we include under abscess?*”  Record 142: “*Any perforation whether contained or not qualifies as complicated*”  Record 208: “*also bleeding*” |
| **Uncomplicated Diverticulitis**- "Acute diverticulitis of diverticulae in the absence of abscess, uncontained perforation, stricture, bowel obstruction, or fistula" | Record 64: “*Microperforation (Contained Perforation) should also be considered "Complicated"*”  Record 124: “*Catastrophic bleeding would then be uncomplicated*”  Record 199: “*I would include both contained and uncontained perforation as complicated. How could a hole in the colon (contained or otherwise) NOT be a complication of the disease? These patients are high-risk and should be appropriately triaged separately from the garden-variety episodic inflammation patient*”  Record 221: “*Would probably just say in the absence of complications as the result of the diverticulitis*” |
| **Chronic Recurrent Diverticulitis**- "Intermittent episodes of acute diverticulitis separated by periods of remission" | Record 24: “*No need for "chronic" in this definition. This is just "recurrent". "Chronic" to me is synonymous with "persistent" and means not getting better or antibiotic dependent*”  Record 172: “*yes, but again need imaging (see a lot of patients who claim they have diverticulitis but the ultimate diagnosis is not diverticulitis)*” |
| **Chronic Persistent Diverticulitis (Smoldering Diverticulitis)**- "Non-remitting symptoms of diverticulitis, including pain, fever, and altered bowel habits" | Record 9: “*I also consider smoldering disease in a patient whose symptoms remit but only briefly*”  Record 143: “*I would want clear evidence of diverticulitis on imaging*”  Record 156: “*prolonged (need a time)*”  Record 167: “*The majority of these patients have some kind of diverticular stricture causing pain rather than a chronic untreatable infection*“  Record 207: “*not always clinically seen and can find endoscopically with no clinical symptoms*” |
| **Symptomatic Uncomplicated Diverticular Disease (SUDD)**- "Persistent or recurrent abdominal symptoms including pain, fever, or altered bowel habits attributed to colonic diverticulosis without signs of overt diverticulitis" | Record 9: “*I would not consider fever to be a feature of SUDD. I'm uncertain about this diagnosis in general*”  Record 24: “*I recognize this disease entity but I view this as "chronic diverticulitis*"”  Record 90: “*I would remove fever from this list as suggests inflammation*”  Record 129: “*If pain and no overt signs of diverticulitis I'm more inclined to say IBS. If add in fever but no overt diverticulitis I am looking for another cause of the fever rather than diverticulosis*”  Record 168: “*SUDD should include people that have intermittent uncomplicated diverticulitis. I would not exclude diverticulitis from this disease classification*”  Record 189: “*Would include need to exclude other causes of those symptoms*”  Record 267: “*No distinction from smoldering diverticulitis*” |
| **Segmental Colitis Associated with Diverticular Disease (SCAD)**- "Nonspecific peri-diverticular inflammation associated with rectal bleeding, abdominal pain, and/or altered bowel habits" | Record 9: “*I would note that the inflammation should spare the rectum. I'm also uncertain that SCAD exists*”  Record 24: “*I believe SCAD is an uncommon intraluminal process distinct from peri colonic diverticulitis so I take issue with the term "peridiverticular" without including "intraluminal"*”  Record 68: “*Too similar to definition of smoldering or chronic recurrent uncomplicated diverticulitis*”  Record 129: “*Think there should be a time course here. Meaning more chronic*”  Record 144: “*I would want to add endoscopy findings to this*”  Record 149: “*Needs to include the absence of IBD*”  Record 184: “*is it non-specific? is the term "peri-diverticular" potentially misleading? Is it more of a "segmental colonic inflammation in the presence of colonic diverticulosis"? Should the element of chronicity of this condition be alluded to in the definition?*”  Record 196: “*Peridiverticular inflammation shouldn't cause rectal bleeding*”  Record 224: “*I do not think it needs to be associated with symptoms. The presence of inflammation alone should be sufficient to define SCAD. Similar to IBD, where patients may not have symptoms, however the fact that there is objective evidence of inflammation should suffice*” |
